# Text Messages Exchanged Between Individuals With Opioid Use Disorder and Their mHealth e-Coaches: Content Analysis Study

**DOI:** 10.2196/37351

**Published:** 2023-03-10

**Authors:** Yerina S Ranjit, Warren M Davis, Andrea Fentem, Raven Riordan, Rikki Roscoe, Patricia Cavazos-Rehg

**Affiliations:** 1 Department of Communication University of Missouri Columbia, MO United States; 2 Department of Psychiatry Washington University School of Medicine in St Louis St Louis, MO United States

**Keywords:** opioid use disorder, opioid, opium, overdose, drug, substance use, content analysis, text message intervention, text message, text messaging, mobile health, mHealth, social support, e-coach, counseling, mental health, depression, recovery support, eHealth, digital health

## Abstract

**Background:**

Opioid use disorder (OUD) has affected 2.2 million people in the United States. About 7.2 million people reported using illicit drugs in 2019, which contributed to over 70,000 overdose deaths. SMS text messaging interventions have been shown to be effective in OUD recovery. However, the interpersonal communication between individuals in OUD treatment and a support team on digital platforms has not been well examined.

**Objective:**

This study aims to understand the communication between participants undergoing OUD recovery and their e-coaches by examining the SMS text messages exchanged from the lens of social support and the issues related to OUD treatment.

**Methods:**

A content analysis of messages exchanged between individuals recovering from OUD and members of a support team was conducted. Participants were enrolled in a mobile health intervention titled “uMAT-R,” a primary feature of which is the ability for patients to instantly connect with a recovery support staff or an “e-coach” via in-app messaging. Our team analyzed dyadic text-based messages of over 12 months. In total, 70 participants’ messages and 1196 unique messages were analyzed using a social support framework and OUD recovery topics.

**Results:**

Out of 70 participants, 44 (63%) were between the ages of 31 and 50 years, 47 (67%) were female, 41 (59%) were Caucasian, and 42 (60%) reported living in unstable housing conditions. An average of 17 (SD 16.05) messages were exchanged between each participant and their e-coach. Out of 1196 messages, 64% (n=766) messages were sent by e-coaches and 36% (n=430) by participants. Messages of emotional support occurred the most, with 196 occurrences (n=9, 0.8%) and e-coaches (n=187, 15.6%). Messages of material support had 110 occurrences (participants: n=8, 0.7%; e-coaches: n=102, 8.5%). With OUD recovery topics, opioid use risk factors appeared in most (n=72) occurrences (patient: n=66, 5.5%; e-coach: n=6, 0.5%), followed by a message of avoidance of drug use 3.9% (n=47), which occurred mainly from participants. Depression was correlated with messages of social support (*r*=0.27; *P*=.02).

**Conclusions:**

Individuals with OUD who had mobile health needs tended to engage in instant messaging with the recovery support staff. Participants who are engaged in messaging often engage in conversations around risk factors and avoidance of drug use. Instant messaging services can be instrumental in providing the social and educational support needs of individuals recovering from OUD.

## Introduction

### Background

Opioid use disorder (OUD) has affected 2.2 million people in the United States. About 7.2 million people report using illicit drugs such as heroin, fentanyl, and prescription opioids, which has contributed to over 70,000 overdose deaths in 2019 [1,2]. This formidable crisis has multiple health and social implications for people with OUD, who are at a high risk of comorbidities, including HIV and mental health disorders, and have higher rates of mortality compared to the general population [3,4]. OUD is also associated with adverse social outcomes including being incarcerated, homelessness, and experiencing social stigma [5-7].

OUD is treated with medication for opioid use disorder (MOUD) using opioid agonist therapy with methadone or buprenorphine [8,9]. MOUD is generally administered over a period of time that involves medically supervised withdrawal, maintenance, and continued psychosocial support for patients [9]. The treatment period differs for each patient depending on the severity of dependence and other medical factors [9]. Behavioral therapy and counseling designed to prevent relapse and support patients are considered the standard of care in addition to pharmacological intervention [9]. Because OUD is a chronic condition that requires pharmacological intervention with ongoing behavioral intervention during recovery, communicating with patients regularly is important to sustain improved health outcomes [9].

Mobile health (mHealth) intervention or the use of mobile phones to improve health holds immense promise in the treatment of chronic conditions including OUD [9-12]. Evidence suggests that mobile phone interventions, including SMS text messaging reminders, have a positive impact on self-management of chronic illnesses [13]. Research shows that patients with OUD spend similar amounts of time on the internet as the general population, and there is high acceptability of mHealth interventions among this population [12,14,15]. A reason that mobile phones are effective in managing chronic illnesses is that users experience a level of social support when information is provided at regular intervals via SMS text messaging [16]. Effectiveness of SMS text messaging programs in initiating treatment as well as preventing relapse has been demonstrated in recent studies [15,17]. Additionally, 70% of the patients at a primary care office-based buprenorphine treatment expressed interest in receiving supportive SMS text messaging, in addition to messages pertaining to the risk of relapse [18]. This suggests there is interest among persons with OUD in receiving social support via mHealth. However, limited studies have examined the content of interpersonal communication between patients in OUD treatment and their providers that have occurred via SMS text messaging.

### Role of Social Support in Patient-Provider Communication in OUD Treatment

Social support occurs when messages, verbal or nonverbal, express directly or indirectly that someone is valued and cared for [19]. Jacobson [20] categorized social support into emotional, cognitive, and material support. Emotional support refers to “behavior that fosters feelings of comfort and leads an individual to believe that he or she is admired, respected, and loved, and that others are available to provide caring and security” [20]. Cognitive support may include information, knowledge, or advice that can help an individual understand their world and adjust to changes, and material support is defined as goods and services that can help solve practical problems [20].

Hence, messages of social support often convey information, emotion, or referral to help someone to manage and reduce uncertainty [16]. Individuals in OUD treatment and recovery experience many uncertainties about the treatment process and outcomes, their personal lives, and social reintegration [21,22]. Additionally, chronic relapse is common in OUD, and research show that social support is crucial for relapse prevention and abstinence [17,23-25]. For example, a study by Polenick et al [26] showed that women undergoing OUD treatment who measured high on loneliness were more likely to start using illicit drugs during recovery compared to those who had greater social support. Additionally, informational support and feeling of closeness played a significant role in the recovery process for pregnant women with OUD and decreased their substance use [27,28]. Hence, there is ample evidence that social support can reinforce the benefits of medication treatments for OUD [29].

Additionally, sustained communication between patients and providers is important for effective treatment outcomes due to the chronic nature of OUD, especially because people with OUD may experience loss of meaningful relationships due to addiction [9,22,24,30]. Supportive relationships between persons with OUD and providers can be defined by mutual trust, respect, and understanding void of prejudice, negative attitudes, or discrimination [9,31]. With the advancement of digital technology, many health interventions effectively use the instant messaging feature on mobile phones to convey messages of social support [32]. The advantages of using SMS text messaging include scalability, relative low cost, and the ability to tailor and personalize messages [32]. While the impact of social support on other health outcomes have been widely examined, social support via mHealth intervention for OUD treatment and recovery has not been well researched. Hence, the purpose of this study is to explore the content of SMS text messaging between participants in a recovery program, with a focus on social support, treatment-related messages, and their relationship with other health outcomes in this population. Understanding social support themes in SMS text messaging exchanges with providers will help us design future text-based mHealth interventions specific to the needs of OUD patients.

### This Study

This study is part of a larger mHealth intervention titled “uMAT-R,” a supplemental support tool to improve adherence to OUD treatment and recovery. The parent study used a mobile app to provide educational content and psychological support for people in OUD recovery programs in the Greater St. Louis Area. uMAT-R also contains modules to support recovery efforts including medication and appointment reminders, and community resources. The primary feature of uMAT-R is the ability for patients to instantly connect with a recovery support staff member or an “e-coach” via in-app messaging. There were 4 e-coaches in this study, who hold bachelor- and master-level degrees in backgrounds including clinical psychology, public health, and social work. e-Coaches received introductory training in person-centered coaching techniques, motivational interviews, patient-centered therapy techniques, and crisis intervention [33,34]. According to the study protocol, after enrollment, the assigned e-coach sent an initial scripted message to let the participants know that they were available for support at any time. If the participants did not respond to the initial message, the e-coach provided a check-in message once a week to let them know that an e-coach was still available. After the initial message, if participants replied to the e-coaches, the response messages from e-coaches were unscripted and tailored to address the specific needs of individual participants. During working hours, e-coaches were tasked with responding immediately after receiving messages from participants. e-Coaches used the first names of the participants while responding to the messages in order to personalize the messages. Each week, e-coaches meet as a team to discuss caseloads and the progress of their clients. They were encouraged to reach out to the project manager or principal investigator whenever needed. If a crisis message was received outside the e-coach’s working hours, the project manager reviewed the message and alerted the e-coach if the message needed to be attended to right away. If the e-coach is unavailable, the project manager or the principal investigator directly responded to the client via messaging or follow-up via phone if there is a concern regarding safety.

This study analyzed these SMS text messages exchanged between participants undergoing OUD recovery and their e-coaches. Additionally, the psycho-educational content within uMAT-R covers topics such as avoiding drug use, dependency, triggers, risk factors, and tips on developing and maintaining healthy alternative habits.

In order to understand the content of these dyadic or two-way messages, this study explores the following research questions: (1) What proportion of in-app social support messages, including messages about emotional, informational, and material support, were exchanged by participants and their e-coaches? (2) What proportion of messages related to the recovery process covered in uMAT-R modules, including messages about avoiding drug use, relapse, triggers, healthy habits, dependency, and risk factors, were exchanged by participants and their e-coaches? (3) How is the mental health condition of OUD recovery participants related to the in-app messaging? (4) What is the nature of dyadic message exchange between participants and e-coaches around topics of social support and OUD recovery?

## Methods

### Sample

For the parent study, participants were recruited from various types of facilities such as OUD outpatient and inpatient programs, recovery homes, hospital settings, medication for addiction treatment clinics, and a clinic that primarily supports pregnant and postpartum women in recovery. Participants were either attending treatment voluntarily or court mandated. Participants were eligible to partake in the uMAT-R mobile app study if they met the following criteria: (1) if they had ever received a formal OUD diagnosis, (2) were currently receiving opioid addiction recovery treatment at one of the above settings, (3) were 18 years or older, (4) were a US resident, (5) were fluent in English, and (6) owned a smartphone with an iOS or Android operating system.

The in-app message exchange between the e-coaches and the participants using the mobile app was retrieved intermittently with the participants’ permission. We retrieved messages over a 12-month period, from 2019 to 2020.

In total, 80 unique dyadic sets of communication occurring between e-coaches and individuals in recovery were identified in the initial data retrieval process, with 1666 individually sent messages present within the dyadic texts. After reviewing the data, the research team removed messages that were unidirectional, such as messages sent by e-coaches without a response from the participants. As a result, the final sample totaled 70 unique dyadic sets, with 1196 individually sent messages. Because the purpose of the study was to examine the impact of the interactive messages, nondyadic messages were excluded from the study. Each dyadic set had a varying number of messages exchanged between OUD patients and e-coaches. The research team aimed to identify the prevalence of thematic elements of social support and OUD-related topics within the data.

### Ethics Approval

This study was approved by the Washington University in St. Louis’ Institutional Review Board (#210805132 and #201910161).

### Statistical Analysis

The prevalence of themes pertaining to social support and topics related to OUD recovery were explored using summative content analysis. In this approach, the research process incorporates identifying and quantifying certain words or thematic elements in a text to understand the context in which they are being used [35]. A summative approach to qualitative content analysis differs from quantitative content analysis in that it goes beyond simply counting words. That is to say, this summative approach includes the process of interpreting content, often referred to as latent content analysis [35]. Additionally, to understand the relationship between mental health outcomes and the nature of the messages exchanged, we conducted a Pearson correlational analysis between individual participant’s scores on depression and anxiety and the type of message (social support and OUD related) sent by each individual.

### Codes

With the primary goal of determining the extent to which messages of social support and OUD-related topics were present in the SMS text messaging interactions, the research team developed a codebook to examine the messages between participants in OUD recovery and their e-coaches. Each case, or individual message, was coded for the following: emotional support messages or messages that foster feelings of comfort and leads the individual to believe that he or she is admired, respected, and loved and that others are available to provide caring and security [36]; cognitive support messages, or informational support, is knowledge or advice that helps the individual to understand his or her world and to adjust to changes within it [36]; and material support messages or messages about goods and services that help solve practical problems [20,36].

The larger intervention aimed to improve the knowledge of individuals who are in recovery around 7 treatment domains. Codes were, hence, created to identify these OUD treatment and recovery domains that included messages of (1) dependency or high level of tolerance for opioid use and a mention of withdrawal symptoms such as diarrhea, sleeplessness, restlessness, irritability, and psychomotor agitation [37]; (2) craving or the desire for more opioid use, as well as the desire to avoid the withdrawal [38]; (3) relapse or recurrence of drug use after a period of abstinence [39]; (4) risk factors or anything that may contribute to use of opioids including alcohol use, psychiatric conditions, home, family, and social environment that will encourage opioid or drug use [39]; (5) triggers or environmental factors, including people or places or moods that trigger drug use [39]; (6) avoidance or self-control and motivation, commitment, and willingness to stay away from drugs, even in difficult situations [40]; and (7) healthy alternative habits or alternatives used in recovery to minimize relapse such as exercising, mindfulness meditation, positive reframing, journaling, and so on [41]. Finally, the codes included mobile app usability issues or problems participants faced that were related to the mobile app.

### Coding Procedure

Each individually sent in-app message served as the primary unit of analysis. This approach allowed us to identify the variable presence on the individual and dyadic level messages. Two coders analyzed the content for either the presence or absence of variables. The training of coders occurred using messages not included in the final coding sample. Both coders engaged in a reliability training over several weeks. In instances of disagreements regarding the interpretation of the thematic content, the research team discussed the concepts in the codebook and compared the data until all discrepancies were resolved. Approximately 173 individual messages were used in reliability training, roughly equating to 11% of the final coding sample, with all variables coded in a binary manner (ie, presence or absence). In coding the final messages sample of 70 dyadic text exchange sets, the research team continued with this process of consensus coding.

## Results

The results showed that 67% (n=47) of the participants in the study identified as female and 33% (n=23) as male. In regards to race and ethnicity, 59% (n=41) were Caucasian, 20% (n=14) African American, and 1% (n=1) Hispanic or Latinx. More than half of the participants reported living in unstable housing conditions and 57% (n=38) were unemployed. See Table 1 for detailed demographic information.

With regard to in-app text messages, out of 70 unique dyadic sets, a minimum of three and a maximum of 87 messages were exchanged between e-coaches and participants. Messages were initiated by e-coaches in most conversations (n=63, 90%), while participants initiated 10% (n=7) of messages. Overall, an average of approximately 17 (SD 16.05) messages per conversation were exchanged. Out of 1196 messages, 64% (n=766) of the messages were sent by the e-coaches and 36% (n=430) were sent by participants. e-Coaches sent an average of 4.1 (SD 5.9) more messages than the participants. Overall, the participants sent an average of 6.6 (SD 7.8) messages per conversation, and e-coaches sent an average of 10.7 (SD 9.4) messages per conversation. The maximum number of messages sent by participants in 1 conversation was 39 and the minimum was 1, and the maximum number of messages sent by e-coaches was 50 and the minimum was 2 per conversation.

**Figure 1:**
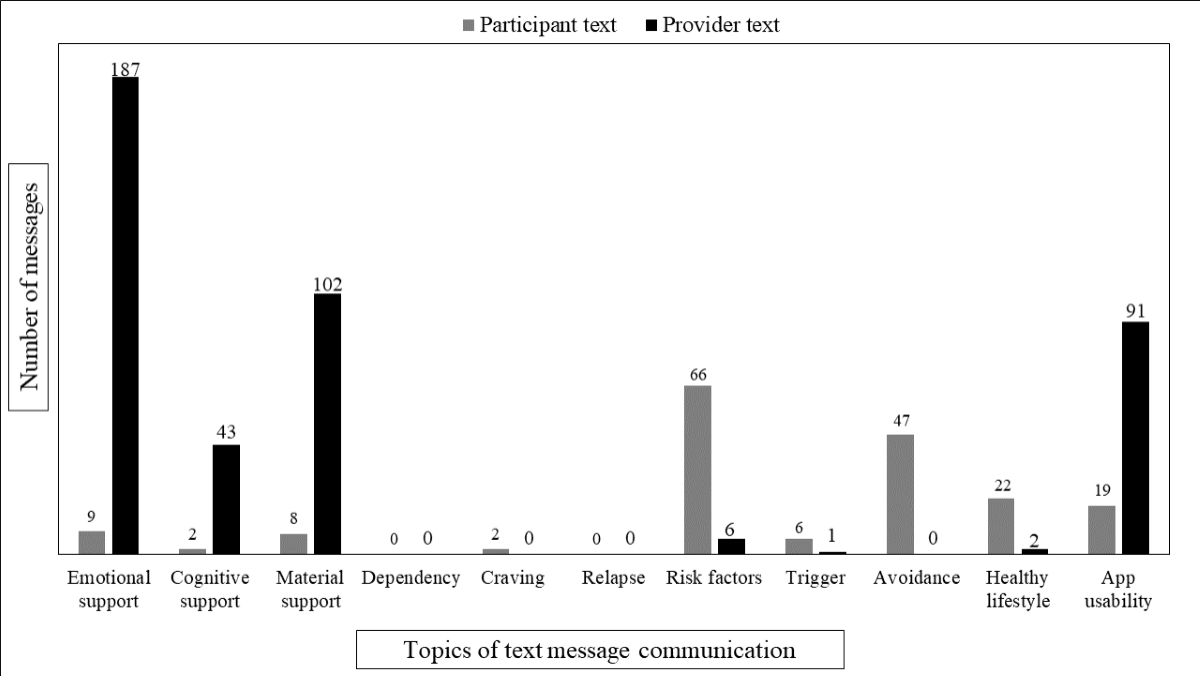
Distribution of 1196 text messages exchanged between patients and providers in the opioid use disorder (OUD) recovery program.

In reference to social support messages, the results showed that emotional support surfaced the most in these dyadic conversations, with 196 occurrences between participants (n=9, 0.8%) and e-coaches (n=187, 15.6%). Material support also occurred at a relatively high frequency, with 110 occurrences between participants (n=8, 0.7%) and e-coaches (n=102, 8.5%). In regard to OUD treatment topics, opioid use risk factors appeared the most with 72 total occurrences, which were mostly sent by participants (n=66, 5.5%) than by e-coaches (n=6, 0.5%), followed by messages of avoidance with 47 total occurrences all sent by patients. Additionally, messages about following a healthy lifestyle were also mostly sent by participants (n=22, 1.8%), with a total of 24 occurrences. Details of message distribution and textual examples are provided in Figure 1 and Table 2.

Additionally, we conducted a Pearson correlation to examine the relationships of the demographic variables, mental health conditions of participants, and adherence to the recovery program to the types of messages presented in Table 3. We found that participants who measured high on depression also sent a higher number of messages of all types of social support (*r*=0.27; *P*=.02). Examining the number of messages only from participants, messages of OUD topics were correlated with messages of social support (*r*=0.42; *P*<.001), and messages of app usability (*r*=0.75; *P*<.001).

Examining the dyadic exchange between participants and e-coaches, we found that messages of social support from e-coaches were highly correlated with messages from participants, especially around messages of social support (*r*=0.34; *P*<.001) and messages around OUD topics (*r*=0.75; *P*<.001).

In Textbox 1, we provide some examples of qualitative data on the communication pattern that support the correlation between individuals with OUD seeking help and e-coach providing support.

**Table 1:** Demographic variables of patients in U-MAT-R program (N=70).

Participant characteristics			Values, n (%)
**Age (years)**			
	19-20	19 (27)	
	31-50	44 (63)	
	51 and older	3 (4)	
**Sex**			
	Male	23 (33)	
	Female	47 (67)	
**Race and ethnicity**			
	Caucasian	41 (59)	
	African American	14 (20)	
	Hispanic/Latinx	1 (1)	
	Other	1 (1)	
**Education**			
	Completed high school	58 (83)	
	College and above	9 (13)	
**Employment**			
	Full-time	16 (23)	
	Part-time	13 (19)	
	Not employed	38 (57)	
**Housing situation**			
	Unstable	42 (60)	
	Stable	25 (36)	
Number of SMS text messaging between participants and e-coach, mean (SD)			17 (16.05)

**Table 2:** Distribution of messages by content category.

Types of messages	Message count (N=1196), n (%)		Sample text
	Participants	e-Coaches	
Emotional support	9 (0.8)	187 (15.6)	Provider: “Hello! I just wanted to check in with you during these times of self-isolation and uncertainty. How have you been doing? Is there anything I can do to help?” Patient: “Hello. Trying to hang in there. It's hard being quarantined, as I'm sure you know. Thanks for asking! I hope you are doing well!”
Cognitive support	2 (0.1)	55 (3.6)	Provider: “Here's a good website for some additional tips on how to manage tantrums as well: https://kidshealth.org/en/parents/tantrums.html?” Patient: “Do you have any medical resources in regards to getting on PrEP?”
Material support	8 (0.6)	102 (8.5)	Provider: “But let me know if you have any questions! You are still able to finish your baseline survey that was sent to your email address and once that's done we can send you your $30 Walmart gift card!” Patient: “thank you for getting back to me. I looked into both of those resources already prior to reaching out. I was looking for additional options.”
Cravings	2 (0.1)	0 (0)	Patient: “I am having really bad Xanax cravings. One of the reasons is my anxiety is really high today.”
Risk factors	66 (5.5)	6 (0.5)	Provider: “Hi there! Just wanted to check in and see how you're doing, I know you've had a very stressful and long week. If you ever want to talk, just know I'm here!” Patient: “MY choices to not let go... how do u let go of someone u kno isnt good for your entire life... but u dnt wanna leave em i dnt kno if it's outta fear or loneliness ... this lifestyle i can't make it”
Trigger	6 (0.5)	1 (0.1)	Provider: “I'm sure that was both emotionally and physically exhausting. I'm glad you're not at work today though and hope you're getting plenty of rest. Triggers like that can be difficult to deal with, no matter how far along you are in your recovery. Acknowledging and processing those triggers the way you did is great, I'm glad to hear you're doing well!” Patient: “hi *****, I'm ok. I'm having a lil issue this week, negative thoughts, not about using but I was in jail last two weeks. things had gone great for nearly two months. since I been home [though] that's changed. i feel I'm still doing great personally, but it seems like things out here went downhill while I was gone. at treatment and here at the house. [There’s] a lot of negativity now and distractions and it has me thinking of changing it up. moving and or switching treatments or at least switching to nights or whatever. i know I could use help or advice, so I thought I'd run it by you. i hope u r doing well.”
Avoidance	47 (3.9)	0 (0)	Patient: “I did my boundaries assignment and I read all the stuff...I got one [haven’t] been the best at setting boundaries but over the years I’ve gotten better.”
Healthy lifestyle	22 (1.8)	2 (0.2)	Provider: “Today’s tip (found on your home screen) talks about a coping plan. Who or what is a part of your coping plan?” Patient: “I did my boundaries assignment and I read all the stuff...I got one haven’t been the best at setting boundaries but over the years I’ve gotten better”
App usability	19 (1.6)	91(7.6)	Provider: “Hi! I wanted to let you know that my coworker [e-coach] will be taking over our messages for a while. Please feel free to reach out to her through the app if you need anything or have any questions moving forward!” Patient: “Just talked with [e-coach] and had to reset my password. now on I tether to get the help I can use. thank you”

**Table 3:** Correlations among the number and types of messages exchanges, mental health, and demographic variables.

		(1)	(2)	(3)	(4)	(5)	(6)	(7)	(8)	(9)	(10)	(11)
**(1) Age**												
	*r*	1										
	*P* value	—^a^										
**(2) Education**												
	*r*	0.18	1									
	*P* value	.14	—									
**(3) Depression**												
	*r*	0.28^b^	0.08	1								
	*P* value	.02	.53	—								
**(4) Anxiety**												
	*r*	0.31^b^	0.05	0.66^c^	1							
	*P* value	.01	.69	<.001	—							
**(5) Adherence to the recovery program**												
	*r*	0.19	−0.07	−0.01	0.02	1						
	*P* value	.14	.59	.96	.89	—						
**(6) Messages of social support from participants**												
	*r*	0.21	0.14	0.27^b^	0.19	−0.06	1					
	*P* value	.09	.28	.02	.12	.64	—					
**(7) OUD** ^d^ **-related messages from participants**												
	*r*	0.13	−0.10	−0.05	−0.05	0.05	0.42^c^	1				
	*P* value	.31	.44	.69	.69	.70	<.001	—				
**(8) Messages of app usability from participants**												
	*r*	0.03	−0.08	0.11	0.12	0.29^b^	0.00	0.06	1			
	*P* value	.79	.51	.36	.30	.02	.97	.61	—			
**(9) Messages of social support from e-coaches**												
	*r*	0.27^b^	−0.05	0.13	0.10	0.14	0.34^c^	0.75^c^	−0.02	1		
	*P* value	.02	.68	.28	.43	.26	.004	<.001	.89	—		
**(10) OUD-related messages from e-coaches**												
	*r*	0.31^b^	0.09	0.11	0.05	0.02	0.10	0.28^b^	−0.09	0.57^c^	1	
	*P* value	.01	.47	.37	.69	.87	.40	.02	.48	<.001	—	
**(11) Messages of app usability from providers**												
	*r*	0.37^c^	0.15	0.09	0.09	0.14	0.19	0.17	0.22	0.28^b^	0.37^c^	1
	*P* value	.002	.24	.46	.46	.25	.12	.16	.07	.02	.002	—

^a^Not applicable.

^b^Correlation is significant at the .05 level (2-tailed).

^c^Correlation is significant at the .01 level (2-tailed).

^d^OUD: opioid use disorder.

**Example text message conversations.**

**Example 1**

- Participant 4: “Today is a bit of a struggle. My husband is working and I’m home alone so lots of things are on my mind.” (Risk factor)
- E-coach: “This is [e-coach’s name], your uMAT-R coach. Thanks for reaching out. What kinds of things are on your mind?” (Responding with emotional support)
- Participant 4: “Just my mind is racing and I keep thinking about old friends I was thinking of reaching out and seeing how they are but I don’t think I should because I don’t know if they are still using or not.” (Need for emotional support and avoidance)
- E-coach: “There are some mediation and relaxation exercises in the courses section of the app that may help your mind calm down a bit. I will set a few goals for you to review them and that may help a bit just to relax. As for wanting to reach out to old friends, it is completely natural to want to catch up and see how they are doing. However, if you are second guessing if it will be a set back to your own health, it may be best to reach out and talk to a friend or family member that you know is supportive in your recovery that does not or no longer uses- this will make you feel a bit more connected to others without putting your own recovery at risk.” (Reaching back with cognitive and emotional support and providing information on healthy alternatives).

**Example 2**

- E-coach: “Hi there! How are you doing today?”
- Participant 71: “So-so. I had my child on [date] but found out I had bleeding on the brain from past [domestic violence] and high BP-- so feeling some type of way about that ...and still staying in a shelter kind [of] wear and tear on me making me having cravings but trying to remain in recovery for me and my child ....just sometimes overwhelmed but waiting wishing I had a someone really for me that I could trust to vent to but also without judgement...but knowing that's not going happen but I'mma keep on ...[I don’t] have a choice.” (Need for social support, healthy alternative, cravings)
- E-coach: “Hi there, I am so sorry to hear that you have been going through all of this. If you need someone to vent to or talk things out with, I am absolutely here for you and I promise to not pass any judgement. You can talk to me about anything you are comfortable with” (Responding with message of emotional support).

## Discussion

### Principal Findings

This study conducted a content analysis of in-app text messages between individuals with OUD and their e-coaches in order to understand social- and OUD-related support needs of this population. Understanding the content of such text-based conversations can be instrumental in guiding the content of future text-based interventions.

The results of this study showed that in this intervention, e-coaches initiated conversation most of the time, but there were instances where the participants were the first ones to send a message. As a part of the design and the protocol of the larger mHealth intervention (uMAT-R), the support staff informed the participants that they would receive their first in-app message from an e-coach, after which they needed to respond to indicate the active functioning of their in-app message feature. The findings also showed that e-coaches sent more messages in each conversation compared to participants, showing that these support staff were actively reaching out and responding to the messages sent by participants. The average number of messages exchanged between e-coaches and the participants showed a healthy trend, without overindulgence.

Further, the results showed that emotional support was the most occurring theme out of the 3 types of social support in the messages. Messages of emotional support from e-coaches occurred the most, followed by messages of material support from e-coaches. Higher number of messages seeking social support from individuals with OUD corresponded with a higher number of support messages sent by e-coaches. Collectively, these findings indicate that the e-coaches tasked with messaging participants reciprocated to the volume of messages sent by participants, a sign of effective interpersonal communication, important for the overall well-being of individuals [42]. While we recognize that studying reciprocity qualitatively was beyond the scope of this study, some qualitative examples of dyadic conversations show that e-coaches often acknowledged the contexts and experiences shared by participants in their response messages, providing evidence for “active listening,” a core value prescribed within the practice of motivational interviewing, for which the e-coaches had been trained prior to engaging in their supportive roles [33,43].

Additionally, the results showed that e-coaches sent a higher number of messages of social support than did the participants. A possible reason for this could be the co-occurrence of multiple themes in a single response from e-coaches. For example, if a participant sent a message about avoiding triggering situations, an e-coach’s message corresponded to the theme of avoidance, which also used a language of reassurance and care that catered to the emotional support need of the participant.

Furthermore, out of several OUD topics, participants sent messages with the theme of risk factors the most, followed by messages of avoiding drug use, possible triggers, and healthy alternative habits. The occurrences of these themes provide evidence for high informational needs due to uncertainties experienced by people in OUD recovery and the presence of various risk factors in their lives despite receiving MOUD, which collectively can contribute to relapse [44]. Hence, recovery and relapse-prevention programs should develop long-term interventions that focus on addressing these informational gaps as well as strategies to reduce perceived and actual risks.

Additionally, this study found that individuals with OUD with mental health needs, specifically those with depression, were more likely to engage in messages of social support. Previous meta-analysis shows that SMS text messaging interventions can have positive impact on managing depression [45]. In sum, because people with depression engage in seeking social support and because SMS text messaging interventions are effective in addressing mental health problems, future interventions should proactively incorporate themes that specifically address the mental health needs of people with OUD.

Overall, this study provides evidence that given an opportunity to engage in web-based two-way communication with their health care providers, people in such OUD recovery programs seek social support. These support needs, as shown in the study, are mostly that of emotional support followed by informational and material support. While measuring the outcome and the impact of the intervention was beyond the scope of this study, it clearly demonstrates that participants continually engage in support-seeking behavior. Programs that employ trained professionals and focus on addressing these needs through consistent communication, not only fill the support need gap but also create a sense of immediacy, defined as “perceived physical or psychological closeness,” which is an important factor in creating trust in providers [46]. Hence, while it is important to address the specific support needs through this web-based intervention, the very presence of a tool that allows for two-way and immediate connection could enhance participants’ perception of support. We recommend future studies to examine these perceptions and their impact further.

### Limitations

While this study provides some important insights into digital intervention for OUD recovery, it is not without limitations. Because this was an exploratory study embedded in a larger parent study, we were not able to include attitudinal measures such as trust in health care providers that could have indicated the participants’ level of trust before and after engaging with the mHealth intervention, an important factor in motivation to complete recovery and abstain from reuse. Future studies should incorporate a larger sample of SMS text messaging for analysis. Additionally, this study could be enhanced by conducting qualitative interviews with participants to understand their motivations as well as facilitators and barriers in using such in-app text messaging services.

### Conclusions

In conclusion, by enumerating the types of social support and OUD topic present in the messages exchanged between people in OUD recovery programs and their support staff, this content analysis provides strong evidence for high social support and relapse-prevention needs of people in OUD recovery. Because of addiction and the need for continuous interpersonal support, text-based messages that are instant, reciprocal, and modeled after principles of effective psychological therapies can be the most cost-effective and sustainable solution to providing long-term support for people recovering from OUD.

## Abbreviations

mHealth: mobile health
MOUD: medication for opioid use disorder
OUD: opioid use disorder

## References

[1] (2023). Trends and statistics: drug overdose death rates. National Institute on Drug Abuse.

[2] (2019). 2019 National survey of drug use and health (NSDUH) releases. SAMHSA.

[3] Larney S, Bohnert AS, Ganoczy D, Ilgen MA, Hickman M, Blow FC, Degenhardt L (2015). Mortality among older adults with opioid use disorders in the veteran's health administration, 2000-2011. Drug Alcohol Depend.

[4] Jones CM, McCance-Katz EF (2019). Co-occurring substance use and mental disorders among adults with opioid use disorder. Drug Alcohol Depend.

[5] Brinkley-Rubinstein L, Zaller N, Martino S, Cloud DH, McCauley E, Heise A, Seal D (2018). Criminal justice continuum for opioid users at risk of overdose. Addict Behav.

[6] Gaeta M, Beitel M, Oberleitner LMS, Oberleitner DE, Madden LM, Tamberelli JF, Barry DT (2020). Correlates of homelessness among patients in methadone maintenance treatment. Med Care.

[7] Perry B, Pescosolido BA, Krendl A (2020). The unique nature of public stigma toward non-medical prescription opioid use and dependence: a national study. Addiction.

[8] Ling W, Wesson DR (2003). Clinical efficacy of buprenorphine: comparisons to methadone and placebo. Drug Alcohol Depend.

[9] (2020). TIP 63: medications for opioid use disorder - executive summary. SAMHSA.

[10] (2020). TIP 63: medications for opioid use disorder - executive summary. SAMHSA.

[11] WHO Global Observatory for eHealth (2011). Mhealth: New Horizons for Health Through Mobile Technologies: Second Global Survey on Ehealth.

[12] Carreiro S, Newcomb M, Leach R, Ostrowski S, Boudreaux ED, Amante D (2020). Current reporting of usability and impact of mHealth interventions for substance use disorder: a systematic review. Drug Alcohol Depend.

[13] Lee JA, Choi M, Lee SA, Jiang N (2018). Effective behavioral intervention strategies using mobile health applications for chronic disease management: a systematic review. BMC Med Inform Decis Mak.

[14] Ranjit YS, Shrestha R, Copenhaver M, Altice FL (2020). Online HIV information seeking and pre-exposure prophylaxis awareness among people who use drugs. J Subst Abuse Treat.

[15] Kmiec J, Suffoletto B (2019). Implementations of a text-message intervention to increase linkage from the emergency department to outpatient treatment for substance use disorders. J Subst Abuse Treat.

[16] Walther JB, Boyd S, Lin CA, Atkin D (2002). Attraction to computer-mediated social support. Communication Technology and Society: Audience Adoption and Uses.

[17] Fishman M, Wenzel K, Vo H, Wildberger J, Burgower R (2021). A pilot randomized controlled trial of assertive treatment including family involvement and home delivery of medication for young adults with opioid use disorder. Addiction.

[18] Tofighi B, Grossman E, Bereket S, D Lee J (2016). Text message content preferences to improve buprenorphine maintenance treatment in primary care. J Addict Dis.

[19] Barnes MK, Duck S (1994). Everyday communicative contexts for social support. Communication of Social Support: Messages, Interactions, Relationships, and Community.

[20] Jacobson DE (1986). Types and timing of social support. J Health Soc Behav.

[21] Muthulingam D, Bia J, Madden LM, Farnum SO, Barry DT, Altice FL (2019). Using nominal group technique to identify barriers, facilitators, and preferences among patients seeking treatment for opioid use disorder: a needs assessment for decision making support. J Subst Abuse Treat.

[22] Brown SE, Krishnan A, Ranjit YS, Marcus R, Altice FL (2020). Assessing mobile health feasibility and acceptability among HIV-infected cocaine users and their healthcare providers: guidance for implementing an intervention. Mhealth.

[23] Zhu Y, Evans EA, Mooney LJ, Saxon AJ, Kelleghan A, Yoo C, Hser Y (2018). Correlates of long-term opioid abstinence after randomization to methadone versus buprenorphine/naloxone in a multi-site trial. J Neuroimmune Pharmacol.

[24] Strang J, Volkow ND, Degenhardt L, Hickman M, Johnson K, Koob GF, Marshall BDL, Tyndall M, Walsh SL (2020). Opioid use disorder. Nat Rev Dis Primers.

[25] Wasserman DA, Stewart AL, Delucchi KL (2001). Social support and abstinence from opiates and cocaine during opioid maintenance treatment. Drug Alcohol Depend.

[26] Polenick CA, Cotton BP, Bryson WC, Birditt KS (2019). Loneliness and illicit opioid use among methadone maintenance treatment patients. Subst Use Misuse.

[27] Fallin-Bennett A, Elswick A, Ashford K (2020). Peer support specialists and perinatal opioid use disorder: someone that's been there, lived it, seen it. Addict Behav.

[28] Asta D, Davis A, Krishnamurti T, Klocke L, Abdullah W, Krans EE (2021). The influence of social relationships on substance use behaviors among pregnant women with opioid use disorder. Drug Alcohol Depend.

[29] Kumar N, Oles W, Howell BA, Janmohamed K, Lee ST, Funaro MC, O'Connor PG, Alexander M (2021). The role of social network support in treatment outcomes for medication for opioid use disorder: a systematic review. J Subst Abuse Treat.

[30] Benville JR, Compton P, Giordano NA, Cheatle MD (2021). Perceived social support in patients with chronic pain with and without opioid use disorder and role of medication for opioid use disorder. Drug Alcohol Depend.

[31] Marchand K, Foreman J, MacDonald S, Harrison S, Schechter MT, Oviedo-Joekes E (2020). Building healthcare provider relationships for patient-centered care: a qualitative study of the experiences of people receiving injectable opioid agonist treatment. Subst Abuse Treat Prev Policy.

[32] Hall AK, Cole-Lewis H, Bernhardt JM (2015). Mobile text messaging for health: a systematic review of reviews. Annu Rev Public Health.

[33] Hettema J, Steele J, Miller WR (2005). Motivational interviewing. Annu Rev Clin Psychol.

[34] Barry MJ, Edgman-Levitan S (2012). Shared decision making—pinnacle of patient-centered care. N Engl J Med.

[35] Hsieh HF, Shannon SE (2005). Three approaches to qualitative content analysis. Qual Health Res.

[36] Langford CPH, Bowsher J, Maloney JP, Lillis PP (1997). Social support: a conceptual analysis. J Adv Nurs.

[37] Spielberger CD (2004). Encyclopedia of Applied Psychology.

[38] Sayette MA, Shiffman S, Tiffany ST, Niaura RS, Martin CS, Shadel WG (2000). The measurement of drug craving. Addiction.

[39] (2020). Drugs, brains, and behavior: the science of addiction. National Institute of Drug Abuse.

[40] Suwanchinda P, Suttharangsee W, Kongsuwan V (2018). Concept analysis: intention to drugs avoidance in adolescents. J Alcohol Drug Depend.

[41] (2022). Addiction as a coping mechanism and healthy alternatives. American Addiction Centers.

[42] Buunk BP, Schaufeli WB (1999). Reciprocity in interpersonal relationships: an evolutionary perspective on its importance for health and well-being. Eur Rev Soc Psychol.

[43] Rollnick S, Miller WR (1995). What is motivational interviewing?. Behav Cogn Psychother.

[44] Luoma JB, Johnson B (2010). Substance use stigma as a barrier to treatment and recovery. Addiction Medicine.

[45] Senanayake B, Wickramasinghe SI, Chatfield MD, Hansen J, Edirippulige S, Smith AC (2019). Effectiveness of text messaging interventions for the management of depression: a systematic review and meta-analysis. J Telemed Telecare.

[46] Richmond VP, McCroskey JC, Hickson M (2008). Nonverbal Behavior in Interpersonal Relations.

